# Exploring MicroRNA Biomarkers for Parkinson’s Disease from mRNA Expression Profiles

**DOI:** 10.3390/cells7120245

**Published:** 2018-12-05

**Authors:** Y-h. Taguchi, Hsiuying Wang

**Affiliations:** 1 Department of Physics, Chuo University, 1-13-27 Kasuga, Bunky-ku, Tokyo 112-8551, Japan; tag@granular.com; 2 Institute of Statistics, National Chiao Tung University, Hsinchu 30010, Taiwan

**Keywords:** biomarker, gene, microRNA, parkinson’s disease

## Abstract

Parkinson’s disease (PD) is a chronic, progressive neurodegenerative disease characterized by both motor and nonmotor features. The diagnose of PD is based on a review of patients’ signs and symptoms, and neurological and physical examinations. So far, no tests have been devised that can conclusively diagnose PD. In this study, we explore both microRNA and gene biomarkers for PD. Microarray gene expression profiles for PD patients and healthy control are analyzed using a principal component analysis (PCA)-based unsupervised feature extraction (FE). 244 genes are selected to be potential gene biomarkers for PD. In addition, we implement these genes into Kyoto Encyclopedia of Genes and Genomes (KEGG) pathways, and find that the 15 microRNAs (miRNAs), hsa-miR-92a-3p, 16-5p, 615-3p, 877-3p, 100-5p, 320a, 877-5p, 23a-3p, 484, 23b-3p, 15a-5p, 324-3p, 19b-3p, 7b-5p and 505-3p, significantly target these 244 genes. These miRNAs are shown to be significantly related to PD. This reveals that both selected genes and miRNAs are potential biomarkers for PD.

## 1. Introduction

Parkinson’s disease (PD), first described by Dr. James Parkinson in 1817, is a chronic, progressive neurodegenerative disease characterized by both motor and nonmotor features [1]. PD motor symptoms such as shaking, rigidity, and slowness of movement are caused by the loss of striatal dopaminergic neurons [2]. The nonmotor symptoms of PD include sleep disorders, depression, and cognitive changes [3,4,5].

The incidence and prevalence of PD increase with age. So far, it is problematic to conclusively diagnose PD because of the lack of a reference standard test [6]. The diagnose of PD is based on a review of patients’ signs and symptoms, and neurological and physical examinations. Resting tremor, cogwheel rigidity, and bradykinesia are three “cardinal signs” of PD, and postural instability, a late finding in PD, is the fourth cardinal sign of PD [6].

Owing to the lack of a standard test for diagnosing PD, genetic testing of mutations in disease-causing genes may be one of a helpful way to diagnose familial Parkinson’s disease (fPD) and sporadic Parkinson’s disease (sPD). A number of PD disease-causing genes have been discovered and debated for both physicians and patients regarding diagnostic and presymptomatic genetic testing of PD in the clinic [7].

A common form of monogenic PD with dominant inheritance is caused by mutations in the gene for leucine-rich repeat kinase 2 (LRRK2) [8,9]; H-Synuclein (SNCA), which is a presynaptic neuronal protein, is linked genetically and neuropathologically to PD [10]; mutations in Parkin are the second most common known cause of PD [11]; mutations in DJ-1 and PTEN Kinase 1 (PINK1) can cause PD [11]; there is a significant inverse association of the ubiquitin carboxy-terminal hydrolase L1 (UCHL1) S18Y variant with PD [12]; and variants in glucocerebrosidase (GBA) and SNCA influence PD risk [13].

In addition to neurological and physical examinations and genetic testing, the gene expression differences between PD and healthy controls can be used as a potential prognosis of PD. Several studies have explored gene biomarkers for PD [14,15]. In this study, we explore gene and microRNA (miRNA) biomarkers for PD.

## 2. Materials and Methods

### 2.1. Gene Expression

We downloaded three mRNA expression profiles of PD and healthy control from Gene Expression Omnibus (GEO) under the GEO ID, GSE20295, GSE20163, and GSE20164. For all three profiles, raw data files (CEL files) (Supplementary file S1) were read with the function ReadAffy of the package Affy (Affymetrix; Thermo Fisher Scientific, Inc., Waltham, MA, USA) in R. Loaded CEL files were treated by mas5 function in the affy package. Then, write.exprs function was used to output normalized mRNA expression profiles. The tissue of the data was substantia nigra (see Supplementary file S1). Integrating the three datasets, we had 35 normal control and 25 PD patients’ substantia nigra mRNA expression profiles.

### 2.2. Principal Component Analysis Based Unsupervised Feature Extraction

We applied principal component analysis (PCA)-based unsupervised feature extraction (FE) to mRNA expression profiles in order to select mRNAs that were expressed distinctly between controls and PD patients (see Supplementary file S2 for more details). This method has been successful in identifying potential biomarkers for other neurological disorders [16,17]. PCA was applied to only mRNAs identified by PCA-based unsupervised feature extraction (FE). PC loadings and PC scores were attributed to samples and genes, respectively. Furthermore, PC loadings are associated with the distinction between controls and PD patients; PD patients and controls were differentiated using linear discriminant analysis (LDA) with these selected PC loadings (see Supplementary file S2 for more details).

### 2.3. Validation of Obtained mRNAs

In order to validate the obtained mRNAs, we computed the area under the curve (AUC) of the receiver operating characteristic curve (ROC). Gene symbols associated with mRNAs selected by PCA-based unsupervised FE were uploaded to Enrichr [18,19], and various enriched biological terms were identified (see Supplementary file S4 for more details).

## 3. Results

255 probes (Supplementary file S3) were identified using PCA-based unsupervised FE. PCA was applied to 255 probes, and PC loadings attributed to samples were computed. The fourth PC loadings turned out to be associated with distinction between PD patients and controls. Table 1 shows the confusion table obtained by the linear discriminant analysis (LDA) using the fourth PC loading. Sensitivity of PD is 0.88, precision of PD is 0.73, F1 score (F-measure) is 0.8, and accuracy is 0.80. AUC is 0.95. Odds ratio is 20.5. *p* value computed by Fisher’s exact test is 2.5 × 10^−6^. All of these evaluations suggest that the fourth PC loadings can significantly discriminate controls and PD patients.

Although we found 255 probes that successfully discriminate PD patients from controls, biological evaluation of obtained probes is important. Two hundred and forty four gene symbols corresponding to these 255 probes (See Supplementary Materials) were uploaded to Enrichr for biological evaluation. There turned out to be many biological terms enriched in these gene symbols. Table 2 shows the top-ranked five categories in “Disease Perturbations from GEO down” of Enrichr (full list is available in Supplementary Material S4). The first four among these five categories are the PD. Since the GEO datasets used in Enrichr are not the same datasets used in our study, PCA based unsupervised FE successfully identified genes downregulated in PD patients.

As for “Disease Perturbations from GEO up” of Enrichr, PD is less enriched, but there are still seven PD experiments with a significant *p*-value (Table 3, full list in Supplementary Material S4).

The next biological term investigated is KEGG pathway (Table 4). Although PD was not top ranked, it is the eighth most significant enriched KEGG pathway. Table 1, Table 2, Table 3 and Table 4 suggest that the identified 244 genes are significantly related to PD.

In this regard, we found that 15 miRNAs significantly target 244 gene symbols by checking “miRTarBase 2017” in Enrichr (Table 5); 113 gene symbols out of 244 gene symbols were targeted by either of 15 miRNAs. These 15 miRNAs were reported to be related to PD (Table 5). Thus, we should consider these 15 miRNAs as key regulators of PD instead of 244 gene symbols. In reality, miRNAs are generally suggested as key regulator of PD [20].

## 4. Discussion

### More Brain Synapse-Related Biological Terms Are Enriched

Although in the previous section, we notice that downregulated genes and upregulated genes in PD GEO datasets and in PD KEGG pathway are enriched in the 244 identified gene symbols, more detailed analyses give us more convincing insight about the relationship between these 244 gene symbols and PD. Other than PD, some KEGG pathways in Table 4 are also related to PD. For example, as for ribosome that is top-ranked in Table 4, ribosomal protein s15 phosphorylation was reported to mediate LRRK2 neurodegeneration in Parkinson’s disease [30] As for phagosome that was the second top-ranked, LRRK2 was reported to be a negative regulator of Mycobacterium tuberculosis phagosome maturation in macrophages [31]. As for synaptic vesicle cycle that was the third top-ranked, synaptic vesicle trafficking was reported to be related to Parkinson’s disease [32]. These results suggest that 244 gene symbols are expected to be closely related to PD progression mechanisms.

Although LRRK2 itself was not included in 244 gene symbols, significant number of genes obtained from “Single Gene Perturbations from GEO up/down” affected by LRKK2 KO/KI (knocked out/knocked in) were included in 244 gene symbols (Table 6 and Table 7). This also supports that 244 gene symbols are related to mechanisms of PD.

Other than PD specificity, it is important to check if 244 genes are specifically upregulated in brain/synapse, since, otherwise, genes identified are not convincing. Table 8 and Table 9 show that 244 genes are highly brain tissue-specific, while Table 10 shows that 244 genes are overlapped with genes downregulated in other tissues than brain (full list is in Supporting Materials).

Thus, we can conclude that 244 gene symbols are not only PD-specific but also brain tissue-specific. This also suggests that we successfully identified gene-related PD mechanisms. In spite of successfully identifications, it is not very easy to investigate as many as 244 gene symbols one by one. It is better to find a more limited number of factors that regulate 244 gene symbols.

For the selected 15 miRNAs in Table 5, we confirmed our results by comparing other studies. hsa-miR-92a appeared as novel hub miR in both regulatory and co-expression network, indicating its strong functional role in PD; GBA deficiency is associated with PD, and miR-16-5p has been shown to correspond to enhanced GBA protein levels [22,33]; and both PD and HD (Huntington’s disease) are neurodegenerative and caused by protein inclusions. miR-615-3p was identified as differentially expressed in HD prefrontal cortex compared to non-neurological disease controls, and hsa-miR-615-3p was identified up-regulated in HD [23]; miR-100, miR-23a, and miR484 were identified to be PD-related miRNAs [25]; miR-320a was identified as a PD-related miRNA [26]; tumor necrosis factor-alpha (TNF-α), a pro-inflammatory cytokine, was elevated in blood, CSF, and striatum regions of the brain in PD patients, and hsa-miR-23a, hsa-miR-23b, and hsa-miR-320a significantly decreased in the presence of TNF-α [27]; mmu-miR-15a-5p, mmu-miR-19b-3p, and mmu-miR-7b-5p miR-7 were shown to downregulate the inflammatory response in cellular in vitro and/or in vivo PD neurotoxic models, and miR-19b was shown to be downregulated in the prodromal stage of alpha-synucleinopathies and pinpointed this miRNA as a potential biomarker also for PD and DLB [20]; and miR-505-3p was identified as a PD-predictive miRNA by microarrays [29].

## 5. Conclusions

PD is a long-term degenerative disorder that usually affects elderly people. Since there are no standard tests that can conclusively diagnose PD, biological biomarkers can help in early diagnosis. In this study, PD-related mRNAs are selected using a PCA-based, unsupervised FE method, and miRNA biomarkers of PD are explored based on these selected mRNAs. Two hundred and forty-four genes and 15 miRNAs are identified to be related to PD. Biological evidence shows the selected genes and miRNAs are potential PD biomarkers. However, since the tissues used in this study are substantia nigra from postmortem brain, it needs to be further verified whether these selected biomarkers can be used as blood/serum PD biomarkers.

## Figures and Tables

**Table 1:** Confusion table obtained by linear discriminant analysis (LDA) using the fourth principal component (PC) loading obtained by principal component analysis (PCA) using 255 mRNAs identified by PCA-based unsupervised feature extraction (FE). Rows: true, columns: prediction.

	Control	PD
Control	24	8
PD	3	22

**Table 2:** Top ranked five categories in “Disease Perturbations from Gene Expression Omnibus (GEO) down” of Enrichr.

Term	Overlap	*p*-Value	Adjusted *p*-Value
Parkinson’s disease DOID-14330 human GSE19587 sample 740	65/207	5.02 × $10^{-83}$	4.18 × $10^{-80}$
Parkinson’s disease DOID-14330 human GSE19587 sample 1080	56/167	5.88 × $10^{-73}$	1.60 × $10^{-70}$
Parkinson’s disease DOID-14330 human GSE19587 sample 496	73/361	3.90 × $10^{-78}$	1.59 × $10^{-75}$
Parkinson’s disease DOID-14330 human GSE7621 sample 940	67/365	2.96 × $10^{-68}$	6.06 × $10^{-66}$
Dystonia C0393593 human GSE3064 sample 329	62/317	1.06 × $10^{-64}$	1.74 × $10^{-62}$

**Table 3:** Seven PD expression profiles in “Disease Perturbations from GEO up” where 244 gene symbols are enriched.

Term	Overlap	*p*-Value	Adjusted *p*-value
Parkinson’s disease DOID-14330 human GSE19587 sample 741	33/158	5.14 × $10^{-35}$	1.22 × $10^{-33}$
Parkinson’s disease DOID-14330 human GSE7621 sample 940	35/235	1.05 × $10^{-31}$	1.74 × $10^{-30}$
Parkinson’s disease DOID-14330 human GSE7621 sample 941	38/342	1.55 × $10^{-29}$	2.19 × $10^{-28}$
Parkinson’s disease DOID-14330 human GSE19587 sample 1080	37/433	1.17 × $10^{-24}$	8.78 × $10^{-24}$
Parkinson’s disease DOID-14330 human GSE6613 sample 788	26/274	1.24 × $10^{-18}$	5.50 × $10^{-18}$
Parkinson’s disease DOID-14330 human GSE19587 sample 496	15/239	1.03 × $10^{-8}$	2.15 × $10^{-8}$

**Table 4:** Top ranked 10 KEGG pathways enriched in 244 identified gene symbols.

Term	Overlap	*p*-Value	Adjusted *p*-Value
Ribosome_Homo sapiens_hsa03010	28/137	1.68 × $10^{-29}$	2.92 × $10^{-27}$
Phagosome_Homo sapiens_hsa04145	16/154	1.72 × $10^{-12}$	1.49 × $10^{-10}$
Synaptic vesicle cycle_Homo sapiens_hsa04721	10/63	4.11 × $10^{-10}$	2.38 × $10^{-8}$
Pathogenic Escherichia coli infection_Homo sapiens_hsa05130	9/55	2.38 × $10^{-9}$	1.04 × $10^{-7}$
Gap junction_Homo sapiens_hsa04540	10/88	1.18 × $10^{-8}$	4.11 × $10^{-7}$
Mineral absorption_Homo sapiens_hsa04978	8/51	2.68 × $10^{-8}$	7.76 × $10^{-7}$
Oxidative phosphorylation_Homo sapiens_hsa00190	10/133	6.09 × $10^{-7}$	1.51 × $10^{-5}$
Parkinson’s disease_Homo sapiens_hsa05012	10/142	1.11 × $10^{-6}$	2.42 × $10^{-5}$
Vibrio cholerae infection_Homo sapiens_hsa05110	6/51	8.84 × $10^{-6}$	1.71 × $10^{-4}$
GABAergic synapse_Homo sapiens_hsa04727	7/88	2.15 × $10^{-5}$	3.73 × $10^{-4}$

**Table 5:** “miRTarBase 2017” in Enrichr when 244 gene symbols were uploaded.

Term	Overlap	*p*-Value	Adjusted *p*-Value	Reference
hsa-miR-92a-3p	37/1404	1.41 × 10^−8^	2.71 × $10^{-5}$	[21]
hsa-miR-16-5p	37/1555	1.93 × 10^−^^7^	1.85 × $10^{-4}$	[22]
hsa-miR-615-3p	25/891	1.38 × 10^−^^6^	8.85 × $10^{-4}$	[23]
hsa-miR-877-3p	19/606	5.92 × 10^−^^6^	2.28 × $10^{-3}$	[24]
hsa-miR-100-5p	12/250	5.37× 10^−^^6^	2.28 × $10^{-3}$	[25]
hsa-miR-320a	18/584	1.33 × 10^−^^5^	4.25 × $10^{-3}$	[26]
hsa-miR-877-5p	11/235	1.68 × 10^−^^5^	4.63 × $10^{-3}$	[24]
hsa-miR-23a-3p	11/249	2.88 × 10^−^^5^	6.91 × $10^{-3}$	[25]
hsa-miR-484	22/890	4.37 × 10^−^^5^	9.33 × $10^{-3}$	[25]
hsa-miR-23b-3p	12/322	6.55 × 10^−^^5^	1.26 × $10^{-2}$	[27]
mmu-miR-15a-5p	15/499	9.42 × 10^−^^5^	1.65 × $10^{-2}$	[25]
hsa-miR-324-3p	12/338	1.04 × 10^−^^4^	1.66 × $10^{-2}$	[28]
mmu-miR-19b-3p	11/310	2.03 × 10^−^^4^	3.00 × $10^{-2}$	[20]
mmu-miR-7b-5p	13/438	3.13 × 10^−^^4^	4.02 × $10^{-2}$	[20]
hsa-miR-505-3p	9/222	2.93 × 10^−^^4^	4.02 × $10^{-2}$	[29]

**Table 6:** Top ranked four LRRK2 KO/KI experiments in “Single Gene Perturbations from GEO up”.

Name	Overlap	*p*-Value	Adjusted *p*-Value
LRRK2 Gly2019Ser (G2019S) mutation knockin human GSE36321 sample 1688	21/335	1.03 × $10^{-11}$	4.82 × $10^{-11}$
LRRK2 mutant human GSE33298 sample 2039	16/309	4.94 × $10^{-8}$	1.55 × $10^{-7}$
LRRK2 dominant negative mutation-G2019S homozygous human GSE33298 sample 1743	12/280	1.68 × $10^{-5}$	4.14 × $10^{-5}$
LRRK2 dominant negative mutation-G2019S homozygous human GSE33298 sample 1741	12/337	1.01 × $10^{-4}$	2.33 × $10^{-4}$

**Table 7:** Top ranked two LRRK2 KO/KI experiments in “Single Gene Perturbations from GEO down”.

Name	Overlap	*p*-Value	Adjusted *p*-Value
LRRK2 Gly2019Ser (G2019S) mutation knockin human GSE36321 sample 1688	24/265	8.38 × $10^{-17}$	9.78 × $10^{-16}$
LRRK2 dominant negative mutation-G2019S heterozygous human GSE33298 sample 1739	9/282	1.61 × $10^{-3}$	3.56 × $10^{-3}$

**Table 8:** Top ranked five gene expression profiles in drug matrix whose altered genes are associated with 244 gene symbols.

Term	Overlap	*p*-Value	Adjusted *p*-Value	
Oxcarbazepine-1600-mg/kg-in_CMC-Rat-Brain-3d-dn	31/369	1.66 × $10^{-20}$		1.31 × $10^{-16}$
Carbachol-15-mg/kg_in_Water-Rat-Brain-3d-up	26/318	4.96 × $10^{-17}$		7.82 × $10^{-14}$
Piracetam-2500_mg/kg_in_CMC-Rat-Brain-5d-up	27/325	7.89 × $10^{-18}$		2.56 × $10^{-14}$
Theophylline-225_mg/kg_in_Water-Rat-Brain-3d-dn	25/314	3.87 × $10^{-16}$		5.08 × $10^{-13}$
Tramadol-114_mg/kg_in_Water-Rat-Brain-5d-dn	26/315	3.93 × $10^{-17}$		7.75 × $10^{-14}$

**Table 9:** Top ranked five gene expression profiles in “Genotype-Tissue Expression (GTEx) Tissue Sample Gene Expression Profiles up” whose altered genes are associated with 244 gene symbols.

Term	Overlap	*p*-Value	Adjusted *p*-Value
GTEX-X585-0011-R2B-SM-46MVF_brain_male_50-59_years	81/1895	1.63 × $10^{-33}$	4.72 × $10^{-30}$
GTEX-WHSE-0011-R2A-SM-3P5ZL_brain_male_20-29_years	71/1660	7.67 × $10^{-29}$	1.11 × $10^{-25}$
GTEX-X261-0011-R8A-SM-4E3I5_brain_male_50-59_years	70/1878	8.35 × $10^{-25}$	8.08 × $10^{-22}$
GTEX-N7MT-0011-R10A-SM-2I3E1_brain_female_60-69_years	70/1918	2.88 × $10^{-24}$	2.09 × $10^{-21}$
GTEX-TSE9-0011-R8A-SM-3DB7R_brain_female_60-69_years	62/1548	2.52 × $10^{-23}$	1.47 × $10^{-20}$

**Table 10:** Top ranked five gene expression profiles in “GTEx Tissue Sample Gene Expression Profiles down” whose altered genes are associated with 244 gene symbols.

Term	Overlap	*p*-Value	Adjusted *p*-Value
GTEX-S4Q7-1226-SM-4AD5I_testis_male_20-29_years	22/329	8.84 × $10^{-13}$	2.36 × $10^{-9}$
GTEX-U4B1-1526-SM-4DXSL_testis_male_40-49_years	20/282	3.48 × $10^{-12}$	3.09 × $10^{-9}$
GTEX-UPK5-1426-SM-4JBHH_liver_male_40-49_years	79/3879	2.06 × $10^{-12}$	2.74 × $10^{-9}$
GTEX-OHPM-2126-SM-3LK75_testis_male_50-59_years	26/525	6.48 × $10^{-12}$	4.07 × $10^{-9}$
GTEX-S7PM-0626-SM-4AD4Q_testis_male_60-69_years	34/911	7.63 × $10^{-12}$	4.07 × $10^{-9}$

## Supplementary Materials

The following are available online at http://www.mdpi.com/2073-4409/7/12/245/s1, File S1: samples for PD and control, File S2: Mathematical details of PCA based unsupervised FE, File S3: the selected genes, File S4: “miRTarBase 2017” in Enrichr when 244 gene symbols were uploaded.
