# Supplementary material for: Exploring MicroRNA Biomarkers for Parkinson’s Disease from mRNA Expression Profiles
Source: Cells. 2018 Dec 5;7(12):245. doi: 10.3390/cells7120245 (PMC6315543; doi:10.3390/cells7120245)
Supplement: Supplementary file 1 [file cells-07-00245-s001.zip › cells-389372-supplementary/cells-389372-SM/cells-389372-wang_supp_S2.pdf]

Supplementary Materials

Y-h. Taguchi, Hsiuying Wang

September 2018

1 Mathematical details of PCA based unsupervised FE

Suppose $x_{ij} \in \mathbb{R}^{N \times M}$ represents expression profiles of ith gene and jth sample. It is also supposed that $\sum_i x_{ij} = 0$ and $\sum_i x_{ij}^2 = N$. The kth $(1 \leq k \leq N)$ principal component (PC) score attributed to ith gene can be obtained as the ith component of the kth eigen vector, $\boldsymbol{u}_k \in \mathbb{R}^N$, of the $N \times N$ matrix XX^T, where X is the $N \times M$ matrix whose component is x_{ij} and X^T is transposition of X, as

$$XX^T \boldsymbol{u}_k = \lambda_k \boldsymbol{u}_k \quad (1)$$

where λ_k is kth eigen values. The kth PC loading attributed to jth sample is the jth sample is the jth component of the kth eigen vector of $M \times M$ matrix, $X^T X$, which can be obtained as

$$\boldsymbol{v}_k = X^T \boldsymbol{u}_k \quad (2)$$

since

$$X^T X \boldsymbol{v}_k = X^T X X^T \boldsymbol{u}_k = X^T \lambda_k \boldsymbol{u}_k = \lambda \boldsymbol{v}_k. \quad (3)$$

After identifying which PC loading attributed samples represent distinction between controls and PD patients, the corresponding PC score used for gene selection. Suppose that the k'th PC loading, $\boldsymbol{v}_{k'}$ are associated with distinction between controls and PD patients, then using the k'th PC score, $\boldsymbol{u}_k$, P-values are attributed to ith gene assuming χ^2 distribution as

$$P_i = P_{\chi^2}\left[> \left(\frac{u_{ki}}{\sigma_k} \right)^2 \right] \quad (4)$$

where $P_{\chi^2}[> x]$ is the cumulative probability of χ^2 distribution when the arugument id larger than x and σ_k is standard deviation. Then P values are adjusted by Benjamin-Hochberg criterion []. Finally, genes associated with adjusted P values less than 0.01 are selected.

2 Practical application of PCA based unsupervised FE

In the following, we introduce how to select 244 gene symbols from gene expression profiles of 32 normal controls and 25 PD patients together with R codes used Suppose that these 57 profiles listed in supplementary files are downloaded into current directory. Then profiles are loaded into R, normalized using `mas5` package as

```
library(affy)
library(methods)
inFilePattern = "*CEL.gz"
outFilePath = "out.matrix"
fileNames = list.files(pattern=glob2rx(inFilePattern))
rawData = ReadAffy(filenames=fileNames)
eset <- mas5(rawData)
write.exprs(eset, file=outFilePath, col.names=NA)
```

Now the file `out.matrix` includes 57 profiles. After gzip this file, we reload this file into R and apply PCA, then identify the sixth PC loading is associated with distinction between control and PD patients,

```
require(readODS)
x <- read.csv("out.matrix.gz",sep="\t")
y <- read.ods("sample.ods",sheet=1)
match(gsub(".CEL.gz","",colnames(x),fixed=T),y[,1])
class <- y[match(gsub(".CEL.gz","",colnames(x),fixed=T),y[,1]),6]
class <- class[-1]
class[class=="control"] <- "Control"
class[class==""] <- "Parkinsons disease"
pca<- prcomp(scale(x[,-1]))
LM <- lm(pca$rotation~class)
SLM<- summary(LM)
fs <- t(data.frame(lapply(SLM,"[",10)))
P0 <- pf(fs[,1],fs[,2],fs[,3],lower.tail=F)
which(p.adjust(P0,"BH")<0.01)
```

since adjusted P values computed by applying t test between controls and samples and are attributed to PC loading is less than 0.01 only for the sixth PC loading. Here sample.ods should be supplementary file that include sample information.

Next, probes are selected using the sixth PC score attributed to genes as

```
P <- pchisq(scale(pca$x[,6])^2,1,lower.tail=F)
index <- p.adjust(P,"BH")<0.01
table(index)
```

This should give us 255 probes. PCA was applied to these 255 probes as

```
pca1 <- prcomp(scale(x[index,-1]))
LM1 <- lm(pca1$rotation~class)
SLM1<- summary(LM1)
fs1 <- t(data.frame(lapply(SLM1,"[",10)))
P1 <- pf(fs1[,1],fs1[,2],fs1[,3],lower.tail=F)
which(p.adjust(P1,"BH")<0.01)
```

Then we found that the only fourth PC loading obtained by re-applying PCA to 244 probes is significantly associated with the distinction between controls and PD patients and is used for linear discriminant analysis (LDA).

3 LDA with PC loading

Using PC loadings re-computed with only 255 probes selected by PCA based unsupervised FE, controls and PC patients are tried to be discriminated by LDA.

```
require(MASS)
LD <- lda(pca1$rotation[,4,drop=F],class,CV=T,prior=rep(1/2,2))
table(class,LD$class)
```

This should results in confusion matrix in the paper (Table **). Here, leave one out cross validation was employed. AUC of ROC was computed as

```
require(caTools)
colAUC(pca1$rotation[,4],class,plot=T)
```

Here `colAUC` function in `caTools` package was used.

4 Conversion to probd ID to gene symbol

Two fundreds fifty five Prob IDs cane be retribed as

```
data.frame(x[index,1])
```

and were uploaded to DAVID. Then gene ID conversion tool wa used to convert probe IDs to gene symbol.

5 Enrichemtn analysis using Enrichr

Gene symbols were uploaded to Enrichr. The results can be accessed via http://amp.pharm.mssm.edu/Enrichr/enrich?dataset=4gev
